# Occlusion and Temporomandibular Function among Subjects with Mandibular Distal Extension Removable Partial Dentures

**DOI:** 10.1155/2010/807850

**Published:** 2010-07-05

**Authors:** N. H. J. Creugers, D. J. Witter, A. Van 't Spijker, A. E. Gerritsen, C. M. Kreulen

**Affiliations:** Department of Oral Function and Prosthetic Dentistry, College of Dental Science, Radboud University Nijmegen Medical Centre, P.O. Box 9101, 6500HB Nijmegen, The Netherlands

## Abstract

*Objective*. To quantify effects on occlusion and temporomandibular function of mandibular distal extension removable partial dentures in shortened dental arches. *Methods*. Subjects wearing mandibular extension removable partial dentures (*n* = 25) were compared with subjects with shortened dental arches without extension (*n* = 74) and with subjects who had worn a mandibular extension removable partial denture in the past (*n* = 19). Subjects with complete dentitions (*n* = 72) were controls. Data were collected at baseline and at 3-, 6-, and 9-year observations. *Results*. Occlusal activity in terms of reported awareness of bruxism and occlusal tooth wear of lower anterior teeth did not differ significantly between the groups. In contrast, occlusal tooth wear of premolars in shortened dental arches with or without extension dentures was significantly higher than in the controls. Differences amongst groups with respect to signs and symptoms related to temporomandibular disorders were not found. Occlusal support of the dentures did not influence anterior spatial relationship. Occlusal contacts of the denture teeth decreased from 70% for second premolars via 50% for first molars, to 30% for second molars. *Conclusions*. Mandibular distal extension removable partial dentures in moderate shortened dental arches had no effects on occlusion and temporomandibular function.

## 1. Introduction

Extension of moderate shortened dental arches (3 to 5 posterior occluding units) is still a controversial issue. The most cited arguments for extending shortened dental arches are improvement of chewing function and rehabilitation of posterior support. Regarding chewing ability, only about 10% of subjects with moderate shortened dental arches reported complaints on chewing function for hard foods [1, 2]. Chewing capacity can be expressed in a scale running from maximum chewing capacity as in complete dentitions to minimum capacity in subjects with full dentures. The chewing capacity of subjects with moderate shortened dental arches is approximately halfway this scale [3]. Moreover, it has been demonstrated that having a moderate shortened dental arch gives no reason for shifts in food selection and does not affect gastrointestinal function dietary [4, 5]. With respect to chewing ability no benefit can be gained from replacing absent teeth unless fewer than three posterior occluding pairs are present [6].

Also the arguments to restore posterior support by distal extension of moderate shortened dental arches remain controversial. It is thought that posterior support prevents or reduces manifestations of the so-called posterior bite collapse. This collapse is accompanied by migrations in the premolar regions, interdental spacing, decrease in vertical dimension, changes in temporomandibular condyle position, overeruption of unopposed teeth, and increased vertical overlap and flaring of anterior teeth [7]. Previous studies showed that in moderate shortened dental arches these phenomenons are just limited or even absent. For moderate shortened dental arches without extension, it was concluded that minor occlusal changes appeared to be self-limiting and adaptive in character [8] and that subjects with shortened dental arches had similar prevalence, severity, and fluctuation of signs and symptoms related to temporomandibular disorders compared to subjects with complete dentitions [9].

This study aims to investigate whether mandibular distal extension removable partial dentures designed for moderate shortened dental arches are beneficial or not to the patients regarding some clinically relevant parameters on occlusion and temporomandibular function.

Metal frame removable partial dentures may generate occlusal interferences by the rests and clasps especially in distal extension types when reduction of the alveolar bone under the saddles is progressing [10, 11]. Although there is no strong evidence for cause-and-effect relationships, occlusal interferences have been regarded as a cause of bruxism [12–15] (i.e. clenching or grinding habits). Occlusal interferences and bruxism have been described provoking further disorders in temporomandibular function [16, 17]. According to this line of reasoning, removable partial dentures have been associated with increased risk for bruxism, and increased risk for signs and symptoms related to temporomandibular disorders [10, 11]. It is assumed that bruxism habits can be verified by occlusal tooth wear as incisal and occlusal wear correlates with bruxism [18]. Occlusal tooth wear has also been associated with the number of teeth, gender, and age [17].

An indicator of increased incisal tooth wear in the anterior region might be a decrease of the vertical overlap. As distal extension removable partial dentures intend to restore posterior support, this decrease of vertical overlap should be even more marked compared to shortened dental arches without these dentures. 

In line with the above arguments regarding occlusion and function, we hypothesized that subjects with shortened dental arches with distal extension removable partial dentures (i) more frequently report awareness of bruxism, (ii) have intensified occlusal tooth wear, and (iii) have more signs and symptoms related to temporomandibular disorders as compared with subjects with shortened dental arches without extension dentures and subjects with complete dentitions. Furthermore, we hypothesized that subjects with extension dentures (iv) have smaller vertical and horizontal overlap of the anterior teeth. 

## 2. Material and Methods

### 2.1. Sample and Observations

For this prospective observational cohort study convenience samples were composed of subjects attending the Nijmegen Dental School Clinic. Three moderate shortened dental arch groups were constructed: (1) subjects with mandibular distal extension removable partial dentures (“SDA + RPD group”); (2) subjects without extension dentures (“SDA group”); (3) subjects without distal extension removable partial dentures but who were wearing this type of denture in the past (“SDA previous RPD group”). As a reference a group of subjects with complete dentitions was constructed (“CD group”). 

A shortened dental arch was classified as moderate if comprising 3 to 5 occlusal units (OUs) with a minimum of 1 OU at each side (left/right) (Table 1). An OU was defined as a pair of occluding natural premolars (1 OU); a pair of occluding natural molars was considered to be 2 OUs. All subjects of the “SDA + RPD group” had a distal extension removable metal frame in the lower jaw; three of them had also this type of denture in the upper jaw. Of the 25 mandibular dentures, 19 were bilateral, and 6 were unilateral distal extension dentures. All dentures (and any replacement denture made during the followup) were conventional removable metal frame dentures without precision attachments and made following the Dental School Clinic protocols. 

Already during baseline observation, most subjects with shortened dental arches had that condition for a long period (see Table 2). Also the removable partial dentures in the “SDA + RPD group” were worn for a long period (see Table 2). Informed consent of the subjects was attained according to the guidelines of the University of Nijmegen. They were interviewed and examined by one calibrated observer at baseline up to 9 years, with 3-year intervals (Table 1). 

Reasons for drop-out were: (1) no further treatment at the dental school clinic for personal reasons (e.g. moving to another area), (2) resignation for other reasons (e.g. not keeping treatment or payment appointments), (3) deceased, and (4) no longer meeting criteria for the dental groups (e.g. due to extraction of teeth). The numbers of dropouts for the dental groups after 9 years for the reasons mentioned were respectively: “SDA + RPD group”: 5, 0, 0, 3; “SDA group”: 12, 9, 1, 3, “SDA previous RPD group”: 2, 1, 0, 4; “CD group”: 18, 8, 2, 3.

### 2.2. Assessments and Scores

Following a structured questionnaire, subjects were asked for awareness of bruxing habits, tempromandibular joint (TMJ) pain/muscle pain/muscle stiffness, restricted mandibular mobility, and clicking/crepitus.

- Bruxism: score 0 = no or not aware; 1 = sometimes; 2 = often.
- TMJ pain: score 0 = no pain; 1 = mild and sometimes pain; 2 = heavy and /or often pain.
- Restricted mandibular mobility: 0 = no; 1 = yes.
- TMJ noises: 0 = no; 1 = yes.

Occlusal tooth wear, vertical and horizontal overlap, maximal mouth opening, TMJ clicking/crepitus and occlusion on denture teeth were assessed clinically:

- Occlusal tooth wear (teeth at right side only): score 0 = no wear facets visible; 1 = facets in enamel; 2 = wear in dentine; 3 = wear in secondary dentine (teeth with artificial crowns were excluded from analysis) [19].
- Vertical and horizontal overlap: measured at the right central incisors by means of a compass and a ruler (in mm).
- Active (unforced) maximal mouth opening (MMO), measured at the right central incisors by means of a compass and a ruler) added by the vertical overlap (in mm): 0 ≥ 45; 1 ≥ 40 and ≤45; 2 ≤ 40.
- TMJ clicking/crepitus: audible or palpable by bilateral palpation during several exercises of opening and closing movements: 0 = no; 1 = dubious; 2 = yes.
- occlusion on denture teeth: recorded in Intercuspal Position using Artus Occlusal Registration strips [20] (13 *μ*m thickness; Artus, Englewood, NJ, USA): yes/no contact with opposing natural tooth. Contacts between mandibular denture teeth and the teeth of the 3 maxillary removable partial dentures were not considered.

### 2.3. Statistical Analyses

For all statistical analyses, a mixed longitudinal model was applied using SAS version 6 software. For “awareness of bruxism” the model (based on a skewed (Poisson) distribution of the scores) analyzed the effects of (1) dental group, (2) time of observation, (3) gender, (4) the period the shortened dental arch existed at baseline observation, and (5) the interaction between observation time and dental group.

For “occlusal tooth wear”, the model (based on a normal distribution of the scores) analyzed the effects of (1) dental group, (2) time of observation, (3) age, (4) reported bruxism, (5) interaction between dental group and time of observation, and (6) interaction between reported bruxism and time of observation. 

For signs and symptoms related to temporomandibular dysfunction, and for anterior vertical and horizontal overlap the model (based on a normal distribution of the scores) analyzed the effects of (1) dental group, (2) time of observation, and (3) interaction between dental group and observation time.

## 3. Results

Mean scores for reported awareness of bruxism (Table 3) revealed a few significant differences amongst the groups. At baseline and at 6-year observation, subjects of the “SDA + RPD group” reported significantly more frequently awareness of bruxism than those of the Complete Dentition group. However, differences within the shortened dental arch groups were statistically not significant except at 6-year observation (“SDA+RPD” more often reported bruxism than “SDA previous RPD”). The mixed model revealed no significant effects on awareness of bruxism for time of observation, gender, nor an interaction between observation time and dental group. The model showed only one significant effect in the ‘SDA previous RPD' the period the shortened dental arch existed at baseline had a significant influence on awareness of bruxism (*P * = .03).

Occlusal wear scores for the lower anterior teeth (Table 4) did not differ significantly amongst the dental groups with two exceptions at the 3-year observation. However, time of observation (*P* < .0001), awareness of bruxism (*P* = .002), and age (*P* = .002) showed significant influence on occlusal wear scores. Also occlusal wear increase over time did not differ significantly amongst the four dental groups. In addition no interaction was found between occlusal wear increase and awareness of bruxism. Occlusal wear scores for the premolars did not differ amongst the three shortened dental arch groups but were significantly higher than in the complete dentition group. The model revealed a significant dental group effect (*P* = .001), observation time effect (*P* < .0001), and age effect (*P* = .02) but no relation between occlusal wear and reported awareness of bruxism. No significant interactions were found. 

Signs and symptoms related to temporomandibular dysfunction were independent from dental group and observation time (Table 5). The only associations found for these variables were interactions between dental group and observation time for the variables restricted mandibular mobility (*P* = .02) and palpated clicking (*P* = .04).

The means of the vertical overlap for the different groups ranged from 2.6 to 3.6 mm, those of the horizontal overlap from 2.7 to 4.0 mm (Table 6). The mixed model demonstrated no dental group effect, but an overall time effect, showing increase of both vertical (*P* < .0001) and horizontal overlap (*P* = .04). 

Regarding occlusion on denture teeth, the more distal the location, the lower the number of occlusal contacts in Intercuspal Position (Table 7). The percentage of potential possible contacts decreased from 70% for second premolars via 50% for first molars to 30% for second molars.

## 4. Discussion

This longitudinal study deals with a relative small convenience sample of subjects with different periods of times that the shortened dental arch existed and different times of wearing or having worn a distal extension removable partial denture at baseline. Matching of the study groups was not perfect. For example, elder subjects with complete dentitions were scarce; consequently the mean age of subjects in the shortened dental arch groups was older than for subjects with complete dentitions. Also, the mean number of occluding units amongst the shortened dental arch groups and gender distribution differed slightly. Nevertheless, we consider the study groups as sufficiently homogeneous to compare with the control group.

Awareness of bruxism was reported in all groups at all observations in almost similar frequencies. We cannot explain the irregular course in awareness of bruxism in the “SDA + RPD group”. A possible explanation could be the relative frequent interventions in this group [2]. Given the relative small sample size, this high incidence could well have affected the outcome, meaning that awareness could be more associated with these interventions rather than with wearing a removable partial denture as such. Moreover, of the 25 subjects with distal extension removable partial denture, five stopped wearing the device during the observation period for various reasons. However, we were not able to substantiate this possible association on the basis of our data. In conclusion, the hypothesis that subjects with moderate shortened dental arches with mandibular distal extension removable partial dentures report more often awareness of bruxism than subjects without such dentures or than subjects with complete dentitions should be rejected. 

On the whole, differences in occlusal wear between the shortened dental arch groups and complete dentitions were only found in the premolar regions. This might be explained by the finding that, although the total maximum bite force of subjects with shortened dental arches is lower compared to that of subjects with complete dentitions [21, 22], the center of occlusal forces has shifted mesially. Consequently, maximal occlusal forces on each individual premolar are higher when molar support is absent [21, 23]. Referring to our hypothesis, as the “SDA + RPD group” did not show significantly more occlusal tooth wear compared to the other shortened dental arch groups, the hypothesis should be rejected. 

Absence of striking differences in occlusal wear in the anterior teeth is in line with the so-called “anterior guidance” phenomenon. In mutually protected occlusions, which are most common in natural dentitions [24], the anterior teeth guide excursions. It seems that this anterior guidance is acting alike in shortened dental arches as well as in complete dentitions. This similar acting also can be denoted from our findings on vertical and horizontal overlap, presenting no relevant differences amongst the dental groups. The latter is not surprising as increased vertical and horizontal overlap—as a symptoms of a posterior bite collapse—is not a matter of excessive loading on maxillary anterior teeth due to reduced posterior support or loss of vertical dimension, rather than resulting from advanced periodontal disease or habits of lips and tongue [25].

Our results regarding reported awareness of bruxism and occlusal tooth wear did not substantiate a possible triggering effect from the distal extension prostheses with respect to temporomandibular related signs and symptoms. 

In general, the distal extension prostheses in moderate shortened dental arches seem to have no positive effects with respect to the investigated occlusal aspects. As such they do not contribute to clinically relevant posterior occlusal support. Restored occlusal support in terms of occlusal contacts by these dentures is just a fraction of that of natural teeth [23], which is confirmed in the present study. The findings of this longitudinal study are in line with those of other studies, suggesting preclusion of making distal extension removable partial dentures in moderate shortened dental arches [6, 26]. If extension of shortened dental arches is considered, evidence indicates that (resin-bonded) cantilever fixed partial dentures is the first treatment option rather than distal extension removable partial dentures [27].

## 5. Conclusions

Within the limitations of this study it can be concluded that subjects with moderate shortened dental arches with or without mandibular distal extension removable partial dentures as compared to subjects with complete dentitions had

(1)similar frequencies for reported awareness of bruxism;

(2)similar occlusal wear of lower anterior teeth; in contrast, premolars had significantly more occlusal tooth wear;

(3)similar frequencies of signs and symptoms related to temporomandibular dysfunction;

(4)no clinically relevant differences of anterior relationships in terms of vertical and horizontal overlap.

Posterior occlusal support by mandibular distal extension removable partial dentures in terms of occlusal contacts in intercuspal position is limited; the more posterior the denture teeth, the less occlusal contacts.

## Figures and Tables

**Table 1:** Number of subjects observed at different followup examinations and age, gender, and number of occlusal units at baseline observation.

	Number (%) of subjects observed at follow-up						
	Baseline	3-year	6-year	9-year	Mean age (SD)	% Female	Mean No (SD) of occlusal units
SDA + RPD	25 (100)	24 (96)	21 (84)	17 (68)	44.1 (8.4)	68	3.5 (0.8)
SDA	55 (100)	47 (85)	41 (74)	30 (55)	40.1 (12.4)	61	3.9 (0.5)
SDA previous RPD	19 (100)	19 (100)	18 (95)	12 (63)	41.7 (9.1)	63	3.4 (0.6)
Complete dentition	72 (100)	59 (82)	53 (74)	41 (57)	36.2 (9.8)	49	12 (0.0)

SDA = shortened dental arch, RPD = mandibular distal extension removable partial denture.

**Table 2:** Number of subjects according to the duration (yr) the shortened dental arch existed at baseline observation.

			Duration (yrs)		
	<5	≥5 and <10	≥10 and <15	≥15	Total
SDA + RPD*	3	6	10	6	25
SDA	13	14	15	13	55
SDA previous RPD	3	4	6	6	19

Total SDA	19	24	31	25	99

*At baseline 9 subjects were wearing an RPD for <5 yrs; 8 subjects ≥5 and <10 yrs; 6 subjects ≥10 and <15 yrs; 2 subjects ≥15 yrs

SDA: shortened dental arch, RPD: mandibular distal extension removable partial denture.

**Table 3:** Mean scores (SD) for reported awareness of bruxism.

	Observations			
	Baseline	3-year	6-year	9-year
SDA + RPD	0.6 (0.7)a**	0.3 (0.6)	0.7 (0.6)b*c*	0.5 (0.6)
SDA	0.4 (0.7)	0.4 (0.6)	0.4 (0.6)	0.5 (0.7)
SDA previous RPD	0.4 (0.7)	0.3 (0.6)	0.2 (0.4)b	0.3 (0.4)
Complete dentition	0.2 (0.4)a	0.3 (0.5)	0.3 (0.5)c	0.4 (0.7)

Same letters indicate significant difference comparing the four dental groups at the same observation.

* : .01 < *P* ≤ .05; ** : .001 < *P* ≤ .01; *** : *P* ≤ .001 SDA: shortened dental arch, RPD: mandibular distal extension removable partial denture.

**Table 4:** Mean scores (SE) of occlusal tooth wear of lower anterior teeth and upper and lower premolars at the right side of the dentition.

	Observations			
	Baseline	3-year	6-year	9-year
Wear on anterior teeth				
SDA + RPD	1.7 (0.1)	1.8 (0.1) a**	1.8 (0.1)	1.9 (0.1)
SDA	1.5 (0.1)	1.7 (0.1)b*	1.9(0.1)	1.9 (0.1)
SDA previous RPD	1.5 (0.1)	1.7 (0.1)	1.8 (0.1)	1.8 (0.1)
Complete dentition	1.4 (0.1)	1.5 (0.1)ab	1.7 (0.1)	1.8 (0.1)
Wear on premolars				
SDA + RPD	1.3 (0.1)c**	1.4 (0.1)e***	1.5 (0.1)h**	1.6 (0.2)k**
SDA	1.0 (0.1)d*	1.2 (0.1)f**	1.4 (0.1)i**	1.4 (0.1)l*
SDA previous RPD	1.0 (0.1)	1.3 (0.1)g**	1.4(0.2)j**	1.6 (0.2)m*
Complete dentition	0.8 (0.1)cd	0.9 (0.1)efg	1.1(0.1)hij	1.1(0.1)klm

same letters indicate significant difference comparing the four dental groups at the same observation.

*: .01< *P * ≤ .05; **: = .001 < *P* ≤ .01; ***: *P* ≤ .001.

SDA: shortened dental arch, RPD: mandibular distal extension removable partial denture.

**Table 5:** Mean scores (SE) for subjective and objective TMD-related signs and symptoms.

	Observations			
	Baseline	3-year	6-year	9-year
Pain				
- SDA + RPD	0.1 (0.1)	0.2 (0.1)	0.2 (0.1)	0.0 (0.0)
- SDA	0.2 (0.0)	0.2 (0.1)	0.2 (0.1)	0.1 (0.1)
- SDA previous RPD	0.2 (0.1)	0.2 (0.1)	0.2 (0.1)	0.1 (0.1)
- Complete dentition	0.1 (0.0)	0.0 (0.0)	0.1 (0.0)	0.1 (0.0)
Reported restricted mandibular mobility				
- SDA + RPD	0.0 (0.0)	0.1 (0.1)	0.1 (0.1)	0.1 (0.1)
- SDA	0.0 (0.0)	0.1 (0.0)	0.1 (0.0)	0.0 (0.0)
- SDA previous RPD	0.2 (0.1)	0.1 (0.1)	0.0 (0.0)	0.0 (0.0)
- Complete dentition	0.0 (0.0)	0.0 (0.0)	0.0 (0.0)	0.0 (0.0)
Reported clicking/crepitus				
- SDA + RPD	0.2 (0.1)	0.2 (0.1)	0.3 (0.1)	0.2 (0.1)
- SDA	0.3 (0.1)	0.4 (0.1)	0.3 (0.1)	0.2 (0.1)
- SDA previous RPD	0.3 (0.1)	0.3 (0.1)	0.1 (0.1)	0.1 (0.1)
- Complete dentition	0.2 (0.0)	0.3 (0.1)	0.2 (0.1)	0.2 (0.1)
Max. mouth opening (mm)				
- SDA + RPD	48 (1)	47 (1)	46 (1)	46 (1)
- SDA	50 (1)	49 (1)	48 (1)	48 (1)
- SDA previous RPD	48 (2)	47 (2)	50 (2)	48 (2)
- Complete dentition	51 (1)	51 (1)	50 (1)	49 (1)
Palpated clicking				
- SDA + RPD	0.2 (0.1)	0.3 (0.1)	0.4 (0.1)	0.3 (0.1)
- SDA	0.2 (0.1)	0.3 (0.1)	0.4 (0.1)	0.1 (0.1)
- SDA previous RPD	0.3 (0.1)	0.3 (0.1)	0.2 (0.1)	0.2 (0.1)
- Complete dentition	0.2 (0.0)	0.2 (0.0)	0.2 (0.0)	0.2 (0.1)

- SDA: shortened dental arch, RPD: mandibular distal extension removable partial denture.

**Table 6:** Mean (SE) vertical and horizontal overlap (mm) at different observation times.

	Observations			
	Baseline	3-year	6-year	9-year
Vertical overlap				
SDA + RPD	2.6 (0.4)	2.9 (0.4)	3.6 (0.3)	3.6 (0.4)
SDA	3.4 (0.2)	3.5 (0.2)	3.5 (0.2)	3.3 (0.3)
SDA previous RPD	3.3 (0.3)	3.4 (0.3)	3.2 (0.4)	3.2 (0.4)
Complete dentition	3.0 (0.2)	3.4 (0.3)	3.4 (0.3)	3.5 (0.3)
Horizontal overlap				
SDA + RPD	2.7 (0.7)	3.4 (0.4)	3.6 (0.3)	3.8 (0.4)
SDA	3.9 (0.6)	3.6 (0.3)	3.8 (0.3)	4.0 (0.4)
SDA previous RPD	3.0 (0.0)	3.4 (0.4)	3.4 (0.4)	3.6 (0.6)
Complete dentition	—	3.5 (0.3)	3.6 (0.3)	3.3 (0.3)

- not measured. SDA: shortened dental arch, RPD: mandibular distal extension removable partial denture.

**Table 7:** Number of occlusal contacts between maxillary natural teeth and opposing denture teeth of the mandibular distal extension removable partial denture. In parentheses the potential number of occlusal contacts (opposing denture teeth present).

	Observations				
Tooth type	Baseline	3-year	6-year	9-year	Combined observations
15/25	8 (11)	6 (10)	7 (8)	5 (7)	26 (36) 72%^a^
16/26	19 (30)	15 (28)	11 (24)	8 (18)	53 (100) 53%
17/27	4 (15)	5 (14)	3 (12)	2 (11)	14 (52) 29%

Total	31 (56)	26 (52)	21 (44)	15 (36)	93 (188) 55%

^a^Percentage refers to the total of recorded occlusal contact and the potential number of contact per tooth type.
